# Valuation of Travel Time Savings in Viewpoint of WTA

**DOI:** 10.1155/2014/305285

**Published:** 2014-11-04

**Authors:** Chang-qiao Shao, Yang Liu, Xiao-ming Liu

**Affiliations:** Key Lab of Transportation Engineering, Beijing University of Technology, Beijing 100124, China

## Abstract

In order to investigate the issues in measurement of value of travel time savings (VTTS), the willingness-to-accept (WTA) for the private car owner is studied by using surveyed data. It is convincing that trip purpose, trip length, time savings, cost savings, income, and allowance from employee have effects on the WTA. Moreover, influences of these variables are not the same for different trip purposes. For commuting trips, effects of income and allowance from employee are significant while time savings and cost savings are dominated for leisure and shopping trips. It is also found that WTA is much higher than expected which implies that there are a group of drivers who are not prone to switching to other trip modes other than passenger car.

## 1. Introduction

The value of travel time savings (VTTS) is one of the critical inputs to transport planning models and tools for management and appraisal of transportation infrastructure investment decisions. Information on VTTS is essential for travel demand models, investment cost-benefit analysis, and road congestion pricing. According to Mackie et al. [1] travel time savings capture 80% of the quantified benefits for transportation cost-benefit analysis. Therefore, various studies were devoted to estimate VTTS for different user types and travel conditions in theory and practice.

With the growing concern for both air pollution and traffic congestion, there is increasing interest in road congestion pricing policy and measuring the total costs of transport modes (i.e., including externality costs along with the direct costs borne by users) in China. Given the importance of VTTS to the congestion pricing, the VTTS must be properly estimated and used and hence study on estimation of VTTS is becoming a more important topic [2]. However, it is hard to obtain the reliable value of VTTS by using the theory method due to three aspect problems. One reason is that there are many people who drive their car not because it is necessary to do but because they love driving (they take driving as a symbol of status and power). Another reason is that there are various invisible allowances for trip such as fuel and parking subsidy and these data are difficult to obtain. The third reason is that many people are nonsensitive to time saving. In order to overcome these problems in exploring the information of VTTS, willingness-to-accept (WTA) is studied.

This paper aims to investigate the issues and variables in measurement of VTTS by analyzing WTA in China. It is organized as follows. The next section reviews the practical and theoretical researches on VTTS, and the following section introduces the used data and method. The influencing variables of WTA and VTTS are presented in Sections 4 and 5.  Section 6 concludes this paper.

## 2. Literature Review

There have been numerous studies on both theory and practice of VTTS since the economic theory about the time allocation was introduced in the 1960s. In neoclassical microeconomics, the VTTS is defined as the willingness-to-pay (WTP) for unit travel time savings. This concept is mainly attributed to Becker [3] and DeSerpa [4]. Becker [3] firstly formalized theory of time allocation and explained how time is valued. He defined the source of utility not as consumption of final goods but as consumption of final commodities. Based on this, Becker's time allocation model was developed and the concept of the VTTS was firstly derived. DeSerpa [4] developed a seminal time allocation model where time spent in different activities is allowed to affect utility in different ways. In the model, the utility maximization problem consists of a budget constraint, a total time constraint, and a minimum time constraint per activity. It is DeSerpa who first stated the distinction between the value of saving time, the value of time as a resource, and the value of time as a commodity. DeSerpa's theory established a firm analytical foundation for value of travel time. Evans [5] incorporated the working time as a direct argument of utility functions; that is, it is stated that working time may be pleasant or unpleasant relative to other activities. Hence, the value of time for all leisure activities was equal and consisted of the wage rate plus the value of working time from the direct utility. Truong and Hensher [6] developed a discrete choice model to estimate VTTS based on the Becker's and DeSerpa's time allocation theory.

The influencing variables (such as trip purpose, level of service, GDP, distance, and saving time) of VTTS are presented and explained in many literatures [1, 7–14]. It shows that the value of time for commuting is greater than leisure [9, 11]. And it is suggested that further research is needed into the effect of the size and sign of the time variation on the estimated value of time [9]. In literature [10], an overview of advances in the valuation of VTTS before 2001 is presented. In this document, the complex influences on evaluation of VTTS are stressed and it is concluded that VTTS is model dependent. Jara-Díaz [11] analyzed effect of individual socioeconomic variables on VTTS and concluded that VTTS is expected to vary with travel and individual socioeconomic environments. Jiang and Morikawa [12] theoretically examined changes of value of travel time savings with travel time, travel cost, wage rate, and work time by using time allocation model for the general case of travel behavior. Axhausen et al. [13] analyzed income and trip distance effect on VTTS across modes as well as across purpose groups. It raises the challenge to current practice in VTTS estimation to move from travel choices to activity choices. Börjesson et al. [14] studied VTTS change over time as incomes grow. They found that the income elasticity of VTTS is not constant but increases with income. Issues such as valuation of working time savings, journey purpose, the mode of travel, journey length, and size of time savings are reviewed by Mackie [1]. It is concluded that direct use of VTTS is inappropriate for social appraisal of projects and that theory cannot tell the relationship between the value of nonworking time and the wage rate while an empirical approach is required.

From the efforts of these researches, it can be concluded that the VTTS are affected by diverse variables and are difficult to estimate. There are still some issues required to be explored.

## 3. Data and Methodology

### 3.1. Data

The data is from a survey about the trip mode choices of passenger car owners. In order to study the effect of congestion pricing on the trip mode choice of the citizens who have private cars, a survey was conducted in Beijing by Beijing Transportation Research Center. A questionnaire was designed and it encompassed two parts. In the first part, each respondent was asked to report the travel mode, trip length, purpose, travel cost, and duration time during the last trip using public transportation. Also, the socioeconomic characters of the travelers such as sex, age, career, and income were included in the first part. In the second part of the questionnaire, diverse congestion pricing scenarios were supposed and for each scenario, available alternative trip modes were listed. The respondents were face-to-face interviewed and asked to fill the questionnaire. Due to the fact that those polled have private cars and most of them prefer to choose passenger car as trip mode, passenger cars are taken as the current trip mode (also it is taken as a faster and more expensive trip mode). If the interviewee changes his or her trip mode on one scenario, we define the chosen mode as the alternative mode (a slower and less expensive mode). The choice of trip mode can be taken as the result of the traveler's trade-off between travel time and travel cost. A total of 3000 respondents are collected.

### 3.2. Methodology

Let us assume that there are only two variables (travel time and travel cost) in the perceived utility and the utility can be written as

(1) $$\begin{array}{c} V_{i}=\beta_{0}+\beta_{1}t_{i}+\beta_{2}c_{i}\;i=1,\ldots ,k, \end{array}$$

where  *V*_*i*_ is utility of choosing travel trip mode *i*; *c*_*i*_ is travel time by using trip mode *i*, CNY; *t*_*i*_ is travel time by using trip mode *i*, minute.

Supposing that the traveler is rational and sensitive to the trip utility, alternative *i* is chosen if and only if *β*_0_ + *β*_1_ *t*_*i*_ + *β*_2_ *c*_*i*_ > *β*_0_ + *β*_1_ *t*_*j*_ + *β*_2_ *c*_*j*_ (*i* ≠ *j*). According to the definition of the VTTS, if an individual prefers alternative *i* to alternative *j*, then his/her VTTS satisfies

(2) $$\begin{array}{c} \hat{\text{VTTS}}=\frac{\beta_{1}}{\beta_{2}}<\frac{c_{j}-c_{i}}{t_{i}-t_{j}}=\frac{\Delta c}{\Delta t}, \end{array}$$

where $\hat{\text{VTTS}}$ is estimate of the VTTS; *c*_*j*_ is travel time for alternative *j*, CNY; *t*_*j*_ is travel time for alternative *j*, minute.

Therefore, according to economic consumer theory [15], if the ratio of travel cost savings with travel time savings is higher than Δ*c*/Δ*t* (Δ*c*/Δ*t* ≥ 0 is required; otherwise, the corresponding interviewee is supposed to not care about travel time and the data will be discarded) the traveler will give up choosing the passenger car as the trip mode; that is, alternative mode (a slower and less expensive mode) will be chosen. In this point, Δ*c*/Δ*t* is the boundary of willingness-to-pay and can be taken as the willingness-to-acceptance (WTA) [16, 17]. Therefore, WTA is defined and calculated as follows:

(3) $$\begin{array}{c} \text{WTA}=\frac{c_{j}-c_{i}}{t_{i}-t_{j}}=\frac{\Delta c}{\Delta t}. \end{array}$$

Although WTA is not the true value of travel time savings, it reflects the information of how much the traveler is willing to pay in order to reduce the travel time. In this point, WTA can be used to describe the characteristics of VTTS and the behavior of individual's trip mode choice. Furthermore, WTA has an advantage over VTTS that VTTS is estimated rather than measured directly while WTA can be measured directly [16]. For these reasons, WTA is analyzed in this paper and is used to explore the character of VTTS.

## 4. Variability of WTA

### 4.1. Effect of Trip Purpose

The surveyed data are classified into different groups according to trip purposes and only three kinds of trip (commuting, shopping, and leisure trips) data are analyzed.  Table 1 summarizes WTA for these three trip purposes. The median values of WTA for commuting, shopping, and leisure are 80.3, 85.3, and 104.8 CNY, respectively. It can be inferred that there are no differences in the median values of WTA for commuting and shopping. However the median values of WTA for leisure are much higher than those for commuting and shopping. Also, from the upper bound of 95% confidence for WTA, it is easily concluded that, for the shopping and leisure trips, the travelers are willing to pay more to save the travel time. This finding is contrary to the conclusion of most literatures [9, 11] that the VTTS for leisure is less than the value for commuting which may be due to the strict requirement of arriving in workplace on time. However, in China, the travelers whose trip purposes are shopping or leisure pay more attention to the trip attributes (such as comfort and convenience), especially for those who are accustomed to travelling with family by passenger cars.

It also can be found that WTA for commuting is left-skewed distribution, while, for the shopping and leisure purpose, WTA is right-skewed distribution. This is not consistent with existing findings [17].

### 4.2. Effect of Time Savings

**Figure 1:**
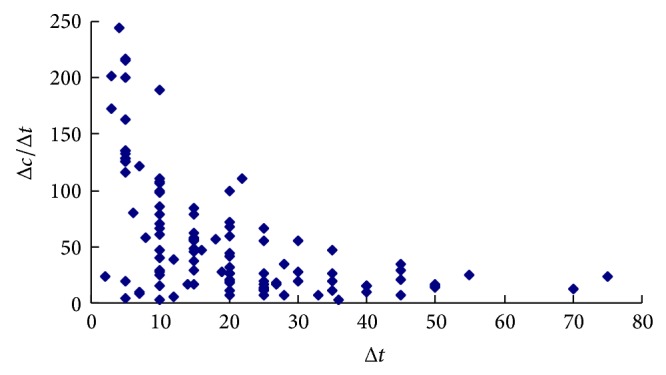
Variability of WTA with time saving for commuting trips.

**Figure 2:**
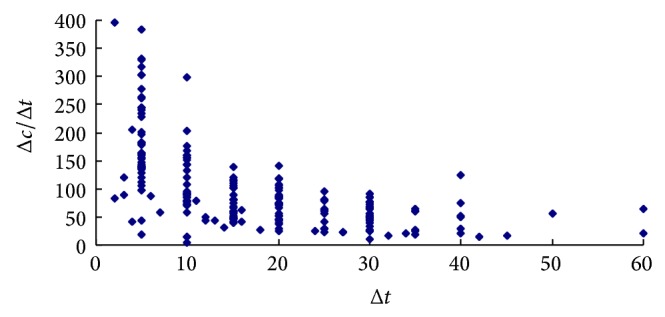
Variability of WTA with time saving for shopping trips.

In theory, VTTS alters with the change of travel time due to the money budget constraint and it has been validated [11]. This implicates that VTTS and WTA should not keep constant with change of time saving size.  Figure 1 shows the relationship of WTA and the time savings for commuting trips. It is illustrated that WTA decreases with the raising of time saving Δ*t*. The same feature is also presented in WTA for shopping trips (see Figure 2).

The value of small time saving is a contentious issue in estimating VTTS [10]. This issue also arises for WTA. Figures 1 and 2 show that WTA is higher than it is expected (for commuting trips, it is higher than 120 CNY/hour and 200 CNY/hour for shopping trips) for small time savings (less than 5 minutes). It can be explained that, for the small time savings, other characters such as comfort and level of service are dominated [16] and that some travelers would not give up driving passenger car.

### 4.3. Effect of Cost Saving

**Figure 3:**
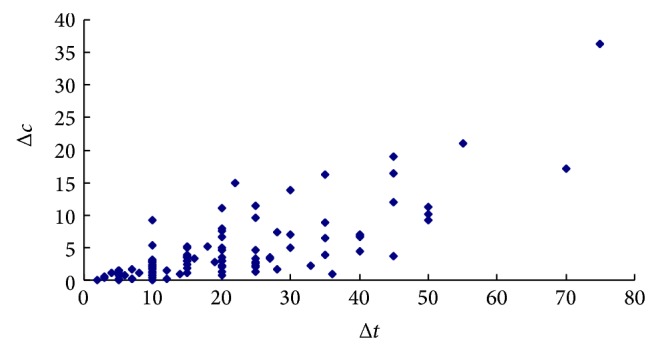
Change of cost saving for commuting trips.

**Figure 4:**
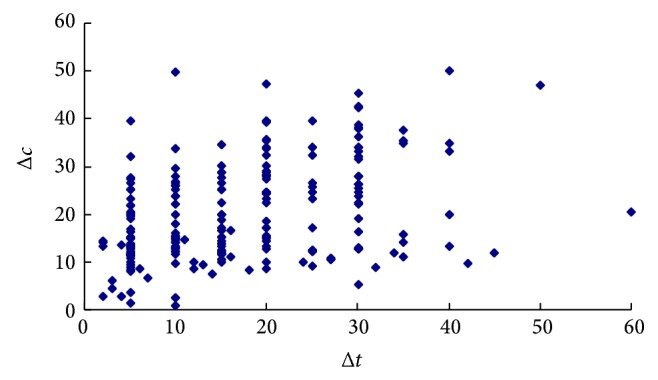
Change of cost saving for shopping trips.

Table 2 lists the statistics of the cost savings for the three kind trips (commuting, shopping, and leisure). From the statistics, it is found that although there are differences among the cost savings, the range of the upper and the lower bound for 95% confidence interval of each kind trip cost saving is very small which means that the cost budget constraint plays a role. Therefore, while the time saving size varies greatly, the cost saving keeps constant (Figures 3 and 4 illustrate the change of cost saving Δ*c* with the time saving Δ*t* for commuting and shopping trips, resp.). It is reasonable that the small time savings are accompanied with higher WTA and WTA decreases with increase of travel time savings.

### 4.4. Discussion of the Results

The effects of variables (e.g., individual income, trip length, trip mode, sex, and career) are discussed in some literatures [1, 7–14]. Therefore, these factors are not analyzed in this paper. This does not indicate that the influences of these variables are unimportant. For this paper, the influences of time saving and cost saving are mainly studied due to the fact that they are often ignored.

## 5. Modelling

A linear model is built to describe the relationship of WTA with the influencing variables. In the model, the trip length, saving time, saving cost, allowance, and individual income are considered. Consider

(4) $$\begin{array}{c} \text{WTA}=\beta_{0}+\beta_{1}t+\beta_{2}c+\beta_{3}\Delta t+\beta_{4}\Delta c+\beta_{5}\text{al}+\sum_{i}\alpha_{i}I_{i}+\varepsilon , \end{array}$$

where WTA is willingness-to-accept, CNY/hour; *t* is trip length measured by the time of car travelled (minute); Δ*t* is travel time saving for choosing more expensive mode (minute); Δ*c* is additional travel cost for choosing more expensive mode (CNY); al = 1, if the traveler gets allowance for this trip; otherwise, al = 0; *β*_*i*_, *α*_*i*_ are parameters; *I*_*i*_ is indicator for income level, using 1, 2, and 3 to indicate low-income, middle-income, and high-income, respectively.

The estimates are presented in Tables 3 to 5. For the commuting (see Table 3), WTA is mainly affected by trip length, trip cost, time saving Δ*t*, cost saving Δ*c*, income level, and allowance. It is confirmed that, with the increase of trip length, WTA decreases. However, it will decrease with the extension of time saving Δ*t*. It is also found that WTA increases with the raise of income. It is interesting to note that allowance has an effect on the WTA for commuting trips while its effect is not significant in the model for leisuretrips and shopping trips.

For leisure trip (see Table 4), WTA decreases with extension of travel time saving Δ*t* while it increases with adding of travel cost saving Δ*c*. It is interesting that income and allowance do not enter into the model. The same conclusion can be made for shopping trip (see Table 5). This implicates that time saving dominates other characters (such as income and trip cost) and that strategic behavior seems to play a role for leisure and shopping trips [16].

## 6. Conclusions and Suggestions

The main contribution of this paper is to extend the analysis of VTTS for these who have passenger cars by studying willingness-to-accept (WTA) and variables for different trip purposes. The analysis results show that WTA is higher than expected which provides evidence suggesting that there are a group of drivers who are not prone to switching to other modes. It is found that both time savings and cost savings are main influence variables which are seldom considered in evaluation of VTTS. It also shows that trip length, trip cost, cost savings, time savings, and income have effects on the WTA for the commuting. However, for the leisure and shopping purpose, only time savings and cost savings are significant in the model which means that these two variables are dominant in mode choice behavior. Another important finding is effect of allowance on WTA which is important in making congestion pricing policy.

In this paper, only parts of influencing variables for WTA of private car owners are studied. Variables such as individual preference in driving and comfort level of service are not mentioned and are remained to be analyzed.

## Figures and Tables

**Table 1:** Summary of WTA for different trip purposes.

Purpose	Statistics of the willingness-to-accept (WTA)						
	Mean	Median	Lower bound	Upper bound	Std. deviation	Skewness	Kurtosis
Commuting	80.3	86.0	64.9	95.7	34.8	.193	−.967
Shopping	96.6	85.3	80.8	101.9	28.9	1.806	2.848
Leisure	106.6	104.8	80.8	132.3	14.3	.996	1.250

**Table 2:** Summary of cost saving for different trip purposes.

Purpose	Statistics of the cost saving						
	Mean	Median	Lower bound	Upper bound	Std. deviation	Skewness	Kurtosis
Commuting	23.4	23.6	20.8	26.0	5.9	−.086	−1.028
Leisure	26.9	27.9	21.9	31.3	10.0	−.260	−.258
Shopping	38.3	36.5	35.7	40.9	14.3	1.095	2.644

**Table 3:** Estimates for commuting trips.

Variable	Parameter estimate	Standard error	*T*	Pr > |*T*|
Intercept	103.388	24.83	6.18	0.0001
*t*	−5.190	0.848	−3.29	0.0002
*c*	−1.863	0.249	−11.069	0.0101
Δ*t*	−2.566	0.249	−11.069	0.0001
Δ*c*	4.422	0.529	8.356	0.0001
*I*_1_	53.452	25.364	1.497	0.0005
*I*_2_	62.551	18.121	1.988	0.0001
*I*_3_	117.218	22.881	5.123	0.0561
al	54.688	17.417	3.140	0.0002

*R* ^2^ = 0.93				

**Table 4:** Estimates for leisure trips.

Variable	Parameter estimate	Standard error	*T*	Pr > |*T*|
Intercept	76.674	16.911	4.534	0.0200
Δ*t*	−4.422	0.627	−7.054	0.0001
Δ*c*	3.993	0.593	6.735	0.0001

*R* ^2^ = 0.87				

**Table 5:** Estimates for shopping trips.

Variable	Parameter estimate	Standard error	*T*	Pr > |*T*|
Intercept	164.120	20.171	66.215	0.0001
Δ*t*	−8.489	0.660	165.231	0.0001
Δ*c*	4.4916	0.562	63.92	0.0001

*R* ^2^ = 0.89				
